# Deisolation in the Healthcare Setting Following Recent COVID-19 Infection

**DOI:** 10.3390/v16071131

**Published:** 2024-07-15

**Authors:** Samuel W. L. Baumgart, Aidan McLachlan, Hayden Kenny, Genevieve McKew, Susan Maddocks, Sharon C.-A. Chen, Jen Kok

**Affiliations:** 1 Department of Infectious Diseases and Microbiology, Concord Hospital, Concord, NSW 2137, Australia; 2 Faculty of Medicine and Health, The University of Sydney, Camperdown, NSW 2050, Australia; 3 Centre for Infectious Diseases and Microbiology, Westmead Hospital, Westmead, NSW 2145, Australia; jen.kok@health.nsw.gov.au; 4 Centre for Infectious Diseases and Microbiology Laboratory Services, Institute of Clinical Pathology and Medical Research, New South Wales Health Pathology, Westmead Hospital, Westmead, NSW 2145, Australia; 5 Centre for Infectious Diseases and Microbiology—Public Health, Westmead Hospital, Westmead, NSW 2145, Australia

**Keywords:** COVID-19, SARS-CoV-2, infection control, deisolation, healthcare, healthcare worker

## Abstract

Background: Deisolation of persons infected with SARS-CoV-2, the virus that causes COVID-19, presented a substantial challenge for healthcare workers and policy makers, particularly during the early phases of the pandemic. Data to guide deisolation of SARS-CoV-2-infected patients remain limited, and the risk of transmitting and acquiring infection has changed with the evolution of SARS-CoV-2 variants and population immunity from previous vaccination or infection, or both. Aims: This review examines the evidence to guide the deisolation of SARS-CoV-2-infected inpatients within the hospital setting when clinically improving and also of healthcare workers with COVID-19 prior to returning to work. Methods: A review was performed using relevant search terms in Medline, EMBASE, Google Scholar, and PubMed. Results and Discussion: The evidence is reviewed with regards to the nature of SARS-CoV-2 transmission, the role of testing to guide deisolation, and the impact of SARS-CoV-2-specific immunity. A paradigm and recommendations are proposed to guide deisolation for inpatients and return to work for healthcare workers.

## 1. Introduction

The COVID-19 pandemic has significantly affected the delivery of healthcare services since the first description of a severe respiratory illness emerged in Wuhan, China in late 2019 [1]. Within healthcare facilities, approaches to the respiratory isolation of patients with COVID-19 need to balance the risk of nosocomial transmission against the impacts of isolation on patient care. Isolation measures would often involve a combination of single or cohorted rooms, and the mandate for increased personal protective equipment (PPE) which must be applied prior to entering the patient’s surroundings.

Isolating patients may have downstream effects on direct patient care including provision of timely investigations and procedures, physical assistance and rehabilitation, patient loneliness, and impact on cognition or critical workflow processes such as increased use of personal protective equipment and waste disposal and altered patient flow and discharge processes [2,3]. Qualitative research in the rehabilitation setting has highlighted patient loneliness, limited group rehabilitative therapy (e.g., gymnasium and hydrotherapy), and even difficulties navigating everyday items within the room (e.g., phone, call button and walker) for patients with physical ailments [2]. More broadly, quarantining in the community has been associated with a higher prevalence of low mood and irritability [4], which is likely to be echoed in inpatient isolation.

The isolation of patients with COVID-19 remains one of several important non-pharmaceutical interventions that assists with reducing the transmission of SARS-CoV-2. Together with hand hygiene and mask use, this reduces the reproduction number (R_0_) by approximately one-third and thus viral transmission [5]. Additional benefits of isolation at the population level were observed in studies from China and New Zealand, demonstrating reduced incidences of other non-SARS-CoV-2 infections, including viral and bacterial respiratory, gastrointestinal, and travel-related infections [6,7,8]. However, there was an observed rise in non-typhoidal salmonellosis and campylobacter-related gastroenteritis cases, which was linked to increased consumption of home-cooked food in one study [8]. These results remain observational, and testing rates may be skewed by changes in health-seeking behaviour compared to pre-pandemic times; however, it is clear that isolation while symptomatic is likely to reduce the spread of infectious diseases.

To balance infection and control measures with the potential costs of isolation, varying deisolation guidelines were published as the pandemic progressed. However, there remains a paucity of clear evidence-based guidance for the deisolation of inpatients within healthcare facilities [9,10]. ‘Deisolation’ encompasses the stepping down from SARS-CoV-2 (irrespective of whether COVID-19 was the primary reason for admission or not)-specific precautions (such as specific PPE and bed allocation) to what would otherwise be required for that patient’s care, i.e., the World Health Organization’s (WHO) ‘Standard Precautions’ for patients not known to be colonised by multi-drug-resistant organisms [11].

The evolution of SARS-CoV-2 variants (including variants of concern [VOCs]), severity of infection in an individual, immunity following vaccination and/or infection, and the use of therapies such as antivirals and monoclonal antibodies may affect the viral kinetics in an infected person and their subsequent risk of transmitting infection [5].

The results of virus culture, nucleic acid amplification tests (NAATs) and rapid antigen tests (RATs) have been used to guide deisolation, despite differences in analytical sensitivity among the three diagnostic methods. Sample type, such as sampling from the lower rather than the upper respiratory tract when pneumonitis is present, may also affect test performance. Further, serial testing, rather than a single test at a specific time point alone, may be more accurate in determining infectivity.

Additional factors including timing since symptom onset, degree of symptom resolution, and underlying immunological status of the affected individual are variably considered within guidelines, and while relevant, contribute to the heterogeneity of the current approaches. In light of these factors, this narrative review provides an overview of viral transmission, testing, immunity, and the current evidence base for deisolation, followed by suggested approaches.

## 2. Objectives and Methods

This review seeks to summarize the literature and expert statements regarding two questions. Firstly, for hospital inpatients improving recovering from SARS-CoV-2 infection, what is the evidence to guide safe deisolation from COVID-19-specific precautions? Secondly, in healthcare workers recovering from SARS-CoV-2 infection, what is the best approach to safely guide return to work? We reviewed contemporary evidence examining COVID-19 transmission, severity of disease, diagnostic testing, and SARS-CoV-2-specific immunity.

**Figure 1:**
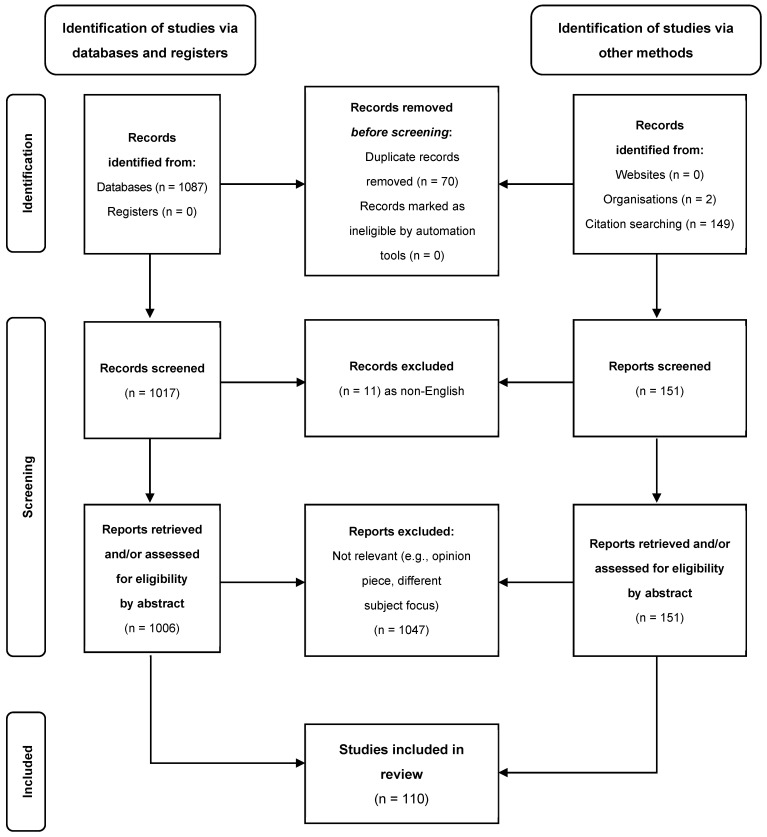
PRISMA diagram for search strategy.

Expert statements from government and international bodies were reviewed between 2020 and 2023, and their reference lists scanned for relevant sources. Medline, PubMed, Google Scholar, and EMBASE were also searched for publications between 2020 and 2023 (including original articles, reviews, and meta-analyses) for “COVID-19”, “SARS-CoV-2”, “testing”, “immunity”, “deisolation”, “inpatient”, “nosocomial transmission”, and related words (as outlined in Figure 1).

Due to the breadth of COVID-19 literature, English-language abstracts were screened by three of the authors independently for relevance and included provided the full-text was available and the discussion was pertinent to inpatient transmission, diagnostics, deisolation, and/or healthcare workers returning to work in patient-facing settings. Articles were excluded on the basis of relevance to the clinical questions above, including editorial letters or reviews with a primary focus on outpatient clinics or wards (e.g., dialysis units, and haematology–oncology outpatients) unless there was no other available evidence from which to extrapolate the data, the latter being highlighted in the text.

## 3. A Brief Summary of SARS-CoV-2 Transmission

Infectious virions of SARS-CoV-2 are now understood to spread from person to person via several routes, primarily via respiratory (airborne/droplet) transmission, as well as through direct contact with infected surfaces or fomites [12]. In their recent narrative review and meta-analysis, Greenhalgh et al. found that airborne transmission is a significant mode of spread of SARS-CoV-2 to alveolar macrophages, the primary site of infection [12]. The authors highlighted several assumptions that underpin the dichotomy between ‘airborne’ and ‘droplet’, namely an arbitrary particle size (<5 μm being considered ‘airborne’), that airborne transmission would result in a higher R_0_, and of note, that the absence of evidence in favour of airborne transmission refutes the possibility of the same [12]. The reader is directed to this article for a further in-depth review, but an understanding of SARS-CoV-2 transmission will in turn impact the type of precautions and therefore deisolation strategies required, whether that includes an ongoing need for mask use, hand hygiene, testing methods for deisolation, and timing of deisolation.

The COVID-19 literature additionally suffers from the varied use of terms used to describe modes of respiratory transmission including ‘airborne’, ‘airborne transmission’, and ‘aerosol’. The WHO has recently revised a new descriptor for pathogens transmitted via the respiratory route as “infectious respiratory particles” (IRPs) [13]. SARS-CoV-2-infected patients may generate IRPs that are expired when speaking, singing, breathing, coughing, spitting or other means, which may then travel through the air and be influenced by environmental factors (such as temperature, air currents, humidity, and sunlight) before potentially reaching another individual [13]. This new definition acknowledges that IRPs exist in a wide range of sizes, and moves away from the ‘airborne’ and ‘droplet’ terminology. Respiratory pathogens may be spread via ‘airborne transmission/inhalation’ or ‘direct deposition’ of IRPs following short-range ballistic movement (e.g., sneezing), which contact the recipient’s mucosal membranes or respiratory system. The report acknowledges that further terminological work is required, and further updates to definitions will be important to acknowledge when providing clearance for deisolation after infection from COVID-19 and a range of other respiratory viruses.

On a practical level, deisolating patients following COVID-19 infection has implications for the type of PPE required for patient care. Traditionally, terms such as ‘airborne’ and ‘droplet’ have differentiated infections on the basis of particle size, and this in turn influences the choice of mask required, perhaps simplistically, to exclude the infectious particle in question. As with the confusion generated by the varied use of such terms, the dichotomy between N95/P2 respirators for ‘aerosol’/‘airborne’ pathogens and surgical/medical masks for ‘droplet’ pathogens is arbitrary, and the same is true of the source of controversy between infection prevention and control clinicians [13,14]. Greenhalgh et al. provided a thorough review of different types of masks and advocate for the use of respirators in the care of COVID-19 patients but acknowledge that surgical/medical and cloth masks do provide some benefit compared to no mask use [12]. In a multi-site noninferiority randomised control trial, Loeb et al. examined rates of COVID-19 infections in healthcare workers caring for COVID-19 inpatients; healthcare workers either wore surgical or N95 masks while caring directly for patients, and all wore surgical masks at other times while within the healthcare facility [15]. Similar rates of RT-PCR-confirmed COVID-19 were observed across both groups after a 10-week follow-up (10.46% for medical masks vs. 9.47% for N95 respirators), suggesting that mask use while caring for COVID-19 patients confers a protective benefit. Notable exclusions included intensive care staff and previously vaccinated or infected individuals. There was also no comment as to the severity of the patients’ COVID-19 illness, which is determined by the presence of upper or lower respiratory tract symptoms, lung infiltrates, and hypoxia (Table 1) [16,17]. This may act as a surrogate of infectious viral load and likelihood of transmission, with prolonged shedding of the infectious virus noted in patients with more severe disease in some studies [18,19].

## 4. Laboratory Testing to Guide Inpatient Deisolation

SARS-CoV-2 was first characterized using metagenomic RNA sequencing of bronchoalveolar lavage fluid collected from a patient with severe respiratory infection [1]. A phylogenetic analysis of the complete viral genome showed that the virus was most closely related to SARS-like coronaviruses [1,6]. The genome was released on 10 January 2020, enabling the development of NAATs and other diagnostic methods, therapeutics, and vaccines. Since then, various diagnostic tests have been employed to guide deisolation, including NAATs by reverse transcription polymerase chain reaction (RT-PCR), viral culture, and RATs. Whilst some laboratory tests can provide information on the amount of replicative (infectious) virus, other factors such as patient symptoms and immune status are also important in determining the risk of transmitting infection [20].

RT-PCR detects SARS-CoV-2 RNA in respiratory samples with high analytical sensitivity and specificity [21]. Depending on the platform and assay utilised, RT-PCR may target one or more viral genes including the open reading frames 1ab (ORF1ab) and 8 (ORF8), nucleocapsid (N), spike (S), envelope (E), transmembrane gene, and RNA-dependent RNA polymerase (RdRp). The relationship of cycle threshold (Ct) values to the infectious viral load and viral culture positivity has been studied, whereby Ct values ≥ 26 or ≥34 predict viral culture negativity [22,23,24]. Whether Ct values below these levels, indicative of a culturable and thus replicative virus, directly correlate with virus transmissibility, is, however, unclear. The use of subgenomic viral RNA detection to determine active viral replication is controversial [25,26] and is not widely available in routine diagnostic laboratories.

Importantly, RT-PCR does not distinguish between non-viable or non-replicating virus and a live or transmissible virus [27]. Non-replicating SARS-CoV-2 may be detected in individuals beyond their infectious period; one meta-analysis suggested a mean duration of shedding of 17 days from upper respiratory tract specimens (maximum 83 days) for mild–moderate disease; however, this is based on data prior to the Omicron VOC and may have altered with changes in population immunity following individual infection and/or vaccination. Older age, male gender, and symptomatic disease are more likely to be associated with prolonged SARS-CoV-2 RNA detection by NAATs without corresponding isolation of live virus [28]. Immunocompromised patients or those with severe disease are likely to shed both live and non-viable virus for longer periods of time compared to immunocompetent patients or those with mild to moderate disease [22,29]. In a case series of immunocompromised patients, Tarhini et al. demonstrated prolonged shedding with RNA detection up to 120 days post-symptom onset, which was associated with the absence of detectable SARS-CoV-2-specific antibodies [29]. In another study of immunocompromised hosts (including B-cell malignancy, receipt of anti-B-cell therapy, and solid organ or hematopoietic stem cell transplant recipients) by Raglow et al., Omicron SARS-CoV-2 was detected by RT-PCR and isolated in 25% and 8% of participants 21 or more days after the initial SARS-CoV-2 detection or illness onset, respectively [30]. Conversely, vaccination is associated with a shorter duration of viral shedding as detected by RT-PCR [31].

Viral culture observing for cytopathic effects and/or substantial increases in the viral load by RT-PCR remains the gold standard for detecting viable virus in respiratory samples. SARS-CoV-2 will not be isolated in the majority of specimens collected from patients with mild to moderate disease by days 8–11 post-symptom onset [20,22,24,32,33,34,35]. By contrast, SARS-CoV-2 may be isolated in patients that are immunocompromised or have severe disease 20 or more days post-symptom onset [20,22]. A small case series of immunocompromised patients on combinations of B- or T-cell-depleting therapies had positive viral cultures up to 103 days post-symptom onset [29]. Numerous authors have attempted to correlate median RT-PCR Ct values from respiratory samples with positive cultures; the upper limit of the Ct values on RT-PCR associated with a positive culture was approximately ≤34, ≤32, 26–34, and ≤27, reflecting the heterogeneity between studies (including but not limited to different patient populations, severity of disease, RT-PCR targets and assays, and virus isolation methods) performed during the different phases of the COVID-19 pandemic with different circulating VOCs [22,23,35,36].

Compared to viral culture and RT-PCR, RATs present a fast and inexpensive testing method that can be used for self-testing or testing at the point of care. Most RATs are lateral flow assays that detect viral antigens, most commonly the nucleocapsid protein [21,27]. A Cochrane review found that compared to RT-PCR, RATs have lower sensitivity for the diagnosis of COVID-19 (73% for symptomatic patients and 54.7% for asymptomatic patients) but retain high specificity > 99% [37]. Despite poor sensitivity for diagnosing infection in patients with lower viral loads (which are typically associated with asymptomatic, pauci-symptomatic, pre-symptomatic, or late infection), several studies have demonstrated that RATs are comparable to viral cultures in identifying patients with a viable (infectious) virus, rendering them a potentially suitable and cost-effective option to help guide the deisolation of patients or allow healthcare workers to return to work [23,38,39,40]. In a prospective cohort study of over 300 patients in the pre-Omicron era (September 2020–January 2021), Lopera et al. showed that negative RATs were equally as effective as viral cultures in detecting individuals who were no longer infectious, with a negative predictive value of 99.9% [23]. Their findings are echoed elsewhere, whereby positive RATs have a high sensitivity for detecting infectious viruses, often correlating with Ct values of ≤25 on RT-PCR [38]. In asymptomatic individuals and those with mild–moderate disease, a negative RAT strongly predicts non-infectiousness [39,40]. Most studies include patients with variable severity of disease and immune compromise; however, dedicated large studies examining RAT vs. culture in severe disease and/or immunocompromised patients are lacking.

Table 2 contrasts the potential advantages and disadvantages of the diagnostic tests for the purposes of deisolation. As further VOCs are expected to emerge over time, it remains pertinent that faster, more readily accessible testing modalities (RT-PCR and RAT) are compared against each other and viral culture for accuracy, sensitivity, and specificity, not only for the initial detection of disease, but also for clinical improvement and the end of viral shedding.

## 5. SARS-CoV-2-Specific Immunity

The measurement of SARS-CoV-2-specific antibodies as a means for determining safe deisolation also attracted significant attention in the earlier phases of the pandemic. The resolution of clinical symptoms has been associated with a declining viral load and an increase in both specific IgG and neutralising antibodies [21]. Seroconversion with antibody titres of at least 1:20 to 1:80 by immunofluorescence was associated with negative viral cultures [34,41]. Despite showing initial promise, the interpretation of serology for SARS-CoV-2 has become more complex with the emergence of new variants of concern, vaccination, hybrid (i.e., from both vaccination and infection) immunity, and increasing number of asymptomatic infections [21,42].

Diagnostic testing for infectious SARS-CoV-2 must now be considered alongside an individual’s immunity to the virus. The immune response to SARS-CoV-2 involves both the innate and adaptive immune systems, the latter involving both humoral and cellular components. Humoral immunity consists of antibodies that typically target the SARS-CoV-2 S protein, and the development of IgG is indicative of prior infection and/or vaccination [43,44,45]. The magnitude of antibody responses following infection generally correlates with the severity of disease. Children with milder disease tend to have lower antibody titres [46,47,48,49], and asymptomatic infection is associated with lower SARS-CoV-2-specific IgG titres [50]. While neutralising antibody responses appear to mitigate against the acquisition of infection, the adaptive humoral and particularly cellular responses are responsible for controlling viral replication after infection and preventing severe disease [51,52]. This will remain relevant in the future as the intersection of new SARS-CoV-2 variants with the complex immune environment (from previous infection and/or vaccine-derived immunity) means that antibody escape rather than innate SARS-CoV-2 virulence becomes the main driver of disease [53]. Furthermore, SARS-CoV-2 antibody levels following vaccination have been shown to wane by a factor of 18.3× over a six-month period, particularly in males and individuals aged greater than 65 years of age [54]. Prospective data by Regev-Yochay et al. suggested antibody level thresholds (>500 binding antibody units/mL or neutralising antibody titres > 1:1024) reduce the risk of infection in household contacts; however, the exact correlation between antibody levels and protection has not been determined [55]. Furthermore, a two- and ten-fold increase in IgG concentrations was associated with an odds ratio of 0.82 (95% CI 0.74–0.92) and 0.43 (95% CI 0.26–0.70) for acquiring infection, respectively [55].

By the end of 2023, there were over 400 SARS-CoV-2 vaccines in development, with 11 granted emergency-use status by the WHO. They were estimated to have saved at least 20 million lives [52]. All approved vaccines have been shown to induce an antibody response at least equivalent to, if not greater than, that induced by previous infection [56,57]. The introduction of SARS-CoV-2 vaccines has markedly reduced disease severity and mortality [58,59].

The currently used SARS-CoV-2 vaccines generate a predominantly IgG antibody response, with less induction of IgA and IgM [60]. Messenger RNA (mRNA) vaccines, such as BNT162b2 (Pfizer-BioNTech), generate a high titre of neutralising antibody that wanes by three to six months with a half-life of approximately 60 days [61,62,63]. The adenovirus vector-based vaccines, such as Ad26.COV2.S, generate lower antibody responses with a slower wane although clinical effectiveness is maintained for at least eight months [64,65,66]. In addition to antibody responses generated by vaccines, an examination of lymph node aspirates from vaccinated individuals suggests durable germinal B-cell and T-cell responses for up to three and six months, respectively, following vaccination [67,68].

Over the course of the pandemic, new VOCs have presented a challenge to vaccine effectiveness. The Omicron variant has over 50 point mutations, compared with four in the Delta variant, and has a fourfold greater immune escape (by neutralising antibody titres) relative to the Beta variant [52,69]. The two-dose primary vaccine regimens confer limited protection against the Omicron variant, and although improved by booster dosing, the protection from infection wanes after four weeks even following four doses [70]. Despite this, T-cell responses remain robust against VOCs, including Omicron, with greater than 80% cross-reactivity [71]. Breakthrough infection from immune-escape SARS-CoV-2 variants is not uncommon; however, robust cellular immunity has translated to retained protection from severe disease, evident in the South African Omicron outbreak where a two-dose primary vaccination schedule provided vaccinees over 70% protection against hospitalisation [72,73].

The titres of neutralising antibody generated from a previous infection are comparable to those following a primary vaccination schedule [74]. Neutralising antibodies and memory B-cells detected by flow cytometry persist for up to eight months in previously infected individuals [57]. One study showed that IgG was still detectable in 87% of individuals ten months post-infection [45]. T-cells responsive to a variety of SARS-CoV-2 viral epitopes were detectable in those previously infected [75]. However, it should be noted that T-cells responsive to the same epitopes were also detectable in pre-pandemic sera and those without previous infection, suggesting the greater promiscuity of T-cells than B-cells [76].

One large meta-analysis by Flacco et al. evaluated SARS-CoV-2 re-infection following primary infection in 15 million subjects aged between 15 and 87 years [77]. At 12 months post-infection, re-infection rates (0.32 vs. 0.77%) and rates of severe disease (0.01% vs. 0.1%) were lower in those who had received at least one dose of a vaccination compared to no doses. The rate of severe or fatal SARS-CoV-2 infection remained low, ranging from 2 to 7 out of 10,000 subjects and did not vary significantly between strains. A similar incremental risk of re-infection following infection rather than vaccination has been shown in a South African study that revealed a 40% relative risk for re-infection with the Omicron variant for those previously infected by the Delta variant, 60% for those previously infected by Beta, and 73% for those previously infected with the original Wuhan strain [78].

Hybrid immunity is most likely to be relevant in the general population currently, given the widespread rates of both previous infection and vaccination. It has been reported that humoral immunity induced by infection is enhanced by vaccination, producing more cross-reactive antibodies targeting the receptor-binding domain of the S protein [79]. Of note, vaccinated persons with acute SARS-CoV-2 infection are more likely to generate a higher serum antibody response, higher nasal mucosal antibody response, and higher S protein-specific CD8+ T-cell response compared to vaccinated individuals without acute infection [80].

Despite its relevance to the cohort of current inpatients, there are no data directly comparing the effect of hybrid immunity versus non-immunity on the onward transmission of SARS-CoV-2 from infected individuals. Of note, an Australian study demonstrated that pooled intravenous immunoglobulin from recent donations maintained the breath of neutralization against previous and emerging SARS-CoV-2 VOCs including the JN.1 variant, but with lower neutralization responses against the newer variants [81]. It is postulated that a higher degree of individual and herd hybrid immunity should reduce the duration of viral shedding; however, in the absence of robust clinical data, any measurement of immunity is unlikely to be incorporated routinely into deisolation guidelines.

## 6. Evidence Base for Deisolating Inpatients within Healthcare Facilities and Recommendations

Healthcare facilities require isolation and deisolation strategies in order to ensure the continuation of healthcare processes while balancing the risk of further infections in the hospital setting. Naturally, the risk of onward infection is not zero and ultimately facilities need to develop a strategy that minimises this risk. That risk may vary between facilities and the patient populations they serve. Interestingly, even public guidelines from government authorities vary for inpatients infected with influenza; isolation or droplet precautions may be mandated anywhere from three days following antiviral therapy to five days without therapy or seven days regardless of therapy [82,83]. The following section seeks to review previously published COVID-19 guidelines and provide a paradigm for use in the inpatient setting, though we acknowledge the framework will need to be adapted to the individual facility.

Despite limited studies examining the efficacy of inpatient deisolation protocols, peak medical bodies including the WHO, United States Centers for Disease Control and Prevention (CDC), and European Centre for Disease Prevention and Control (ECDC) have used available surrogate evidence to provide medical facilities with consensus statements about patient deisolation [9,10,17]. Key studies are highlighted in Table 3. Early guidelines mandated clinical recovery and two negative RT-PCR tests collected at least 24 h apart; however, improved knowledge around prolonged viral shedding and RT-PCR positivity, viral kinetics (which may be affected by the receipt of antiviral therapies), and persisting symptoms such as dry cough called this into question. Subsequent 2020 WHO guidelines suggested a time-based approach from symptom onset, whereby asymptomatic patients could be deisolated after ten days without testing, and those who were symptomatic required an additional three days of being symptom-free following their ten-day period of isolation [17]; however, such predominantly time-based strategies fail to acknowledge that an individual’s immune status may influence viral shedding. More recent 2022 and 2023 guidelines [9,10,84] have adopted the nuances provided by an understanding of testing, viral kinetics, and immunity, often incorporating RAT-led deisolation testing. Despite updates to guidelines, there is a paucity of data demonstrating the risk of nosocomial transmission or onward transmission from patients who have been deisolated following an acute COVID-19 illness; the absence of which may promisingly point towards current guidelines being appropriately conservative [85].

The severity of clinical disease has been associated with prolonged viral clearance and infectivity, alongside comorbidities such as hypertension, chronic obstructive pulmonary disease, and hyperlipidemia [19,91,92]. Varying evidence also exists regarding whether more rapid viral clearance occurs in asymptomatic patients versus symptomatic patients; however, a recent meta-analysis by Wu et al. suggests no significant difference exists [32,93,94]. As such, asymptomatic and mild–moderate infections are often grouped similarly in guidelines.

In asymptomatic, mild, and moderate COVID-19 infections, patients are most contagious immediately prior to the onset of symptoms or a positive test and the first few days following symptom onset [95]. A consistent finding in the literature is that this cohort is unlikely to be infectious beyond day ten from symptom onset, and this is reflected in international guidelines where patients may be deisolated in healthcare settings at day ten without fulfilling any testing criteria [9,10,20,28,84,95,96]. Guidelines vary in their inclusion of vaccination status in deisolation practices, particularly given the widespread infection rates that occurred internationally with the Omicron VOC, hybrid immunity, and minimal effect on test positivity [88,97,98].

Several studies incorporate symptom onset, resolution, testing strategies, and mask wearing in the deisolation of patients with asymptomatic, mild, or moderate disease. Bays et al. found that the use of RATs on days six and seven for patients would allow 79% of patients to be deisolated by day seven from symptom onset [89]. Their findings are supported by Maya and Kahn, who in a modelling study with the Omicron VOC found that a negative RAT on day six of a ten-day total isolation allowed for a cost-effective approach to deisolating patients, with a low rate of secondary infections, compared to RAT testing on day five of an eight-day total isolation, RT-PCR testing on day five of a ten-day isolation, or the 2022 CDC guidance to perform a symptom check on day five of a ten-day isolation [9,99]. These findings are supported by the high negative predictive value (99.5%) of RATs in ruling out infectious patients [100]. A combined testing- and symptom-based approach is adopted by several guidelines, in which the resolution of symptoms by day five or six and a negative RAT would allow for earlier deisolation [10,84]. Clearly, RATs provide a rapid, point-of-care test with utility in this setting, but their use should be combined with symptom improvement and time since onset of illness to improve their accuracy in guiding deisolation.

The use of RT-PCR for deisolation is incorporated in some international guidelines, and typically includes the requirement of a negative test result [9,10]. A negative result may thus be helpful in deisolation protocols, but a positive RT-PCR test result in immunocompetent asymptomatic patients is more difficult to interpret and should not be routinely recommended. The recommendation for RT-PCR in some guidelines does not consider that RT-PCR test results with Ct values >30–32 are generally not associated with a viable virus and errs on the side of caution given the variability between Ct values across the different RT-PCR assays due to variable targets and the potential for the late detection of drop-out targets such as the S gene and N gene for the B.1.1.7 and BA.2.86 SARS-CoV-2 variants, respectively [24,27,36,90]. Additionally, as most RT-PCR assays are qualitative or semi-quantitative, Ct values should be interpreted with caution unless a validated quantitative assay with an understanding of the viral load is supported in that particular institution [101,102].

**Recommendation:** In light of the above, for asymptomatic, mild, or moderate disease in non-immunocompromised patients, regardless of vaccination status, deisolation should be considered if the patient is asymptomatic by day five and has either a negative RAT or RT-PCR test on day six or seven. If SARS-CoV-2 RNA is detected by RT-PCR, but with a Ct value ≥ 30, a RAT should be performed and the decision to deisolate determined by the RAT result. In the absence of further testing, ten days of isolation should be observed (Table 4).

The decision to deisolate patients with severe or critical COVID-19 disease or immunocompromised patients is more nuanced and challenging. Evidence is limited to smaller cohorts and should be interpreted with caution. As previously noted, viral shedding can occur for prolonged periods in these patient cohorts, and a more individualised approach should focus on symptom resolution, allowing for at least 20 days since symptom onset, and two negative tests (RT-PCR test or RAT) collected 24 h apart. In the critical care setting, consensus guidelines recommend, without substantial evidence, a 20-day minimum isolation period, but if bed availability were to become an issue, a ten-day minimum isolation period followed by two negative RT-PCR tests may be considered [103]. In the haemodialysis population, it has been recommended that RT-PCR Ct values and/or RAT be considered in guiding deisolation, in keeping with viral kinetics studies in immunocompromised patients [104]. Despite the paucity of data regarding onward infection after deisolation, Kim et al. reported the case of a renal transplant recipient who was isolated for ten days with clinical recovery after a COVID-19 infection with the Delta VOC, only to require pulsed corticosteroid therapy for transplant rejection. This patient subsequently became symptomatic of COVID-19 and infected a number of other patients, carers, and healthcare workers. The same strain was confirmed on whole genome sequencing (WGS), suggesting a reactivation after immunosuppression following deisolation [105]. Therefore, the recommendation for deisolation in this setting should take into consideration persistence or recrudescence of symptoms, time elapsed since symptom onset, testing results, and plans for future immunosuppression but also the availability or use of prolonged or repeated courses of anti-viral medications.

**Recommendation:** Deisolation following severe or critical disease should be guided by a combination of symptom duration and testing results. For those who have improved symptomatically, a negative RAT or RT-PCR test after day ten may guide deisolation; however, deisolation could be considered at 20 days post-symptom onset in the absence of testing. (Table 4).

**Recommendation:** Deisolating immunocompromised patients should be individualised. Acute COVID-19 symptoms should have completely resolved, and respiratory parameters should be improving before deisolation is considered. A combination of at least 14 days post-symptom onset and two negative RT-PCR tests or RATs taken 24 h apart provide confidence to deisolate the patient. Patients should limit their exposure to high-risk settings (hospital appointments and aged care facilities) and consider wearing a surgical mask until day 20 post-symptom onset (Table 4).

## 7. Evidence Base for Deisolating Healthcare Workers and Recommendations

In the early phases of the pandemic, zero-tolerance COVID-19 isolation strategies of at least two weeks’ isolation and mandatory negative NAATs prior to release were widely acknowledged to have effectively reduced morbidity and mortality, but at significant financial costs and impact on the medical workforce affecting patient care and patient mental health [106].

Since early 2022, the surge of the Omicron VOC as the predominant community SARS-CoV-2 strain and further knowledge about NAAT interpretation and viral kinetics have contributed to a transition away from more conservative deisolation strategies for HCWs towards shorter time periods and revisiting the need for a negative RT-PCR test or RAT. Although Omicron was more infectious than the previous Delta and Alpha VOCs, it had a significantly shorter period of infectivity, justifying a shortened isolation period [97,98,107,108]. This shortened period of infectivity has been demonstrated in multiple studies using viral culture as a surrogate measure of infectivity in SARS-CoV-2 Omicron patients [32,91,92].

Guidelines for HCWs returning to work following COVID-19 infection currently recommend a minimum of five days of isolation, with return to work on day six post-symptom onset following a negative RAT or day seven without any testing, provided the HCW is asymptomatic [9,84]. The most significant difference between these and earlier guidelines is the management of close or suspected COVID-19 exposures, with current guidelines promoting continuing to work and risk minimisation strategies such as wearing masks in clinical areas until day ten rather than a prescribed duration of quarantine. RATs are the preferred method of deisolation, given their comparable sensitivity and specificity, up to 96.2% and 91%, respectively, at a significantly lower cost and shorter turnaround times compared to RT-PCR tests [39,99]. As discussed, RATs correlate well with infectivity, including for Omicron VOCs.

A larger retrospective study by Khan et al. (n = 1340) looked at the effectiveness of implementing a modified return to work policy similar to current Australian guidelines [86]. In this model, HCWs performed RATs between days five and seven of illness and were allowed to return to work the day following a negative result. This contributed to 3799 additional days of work saved compared to a standardised isolation period of ten days with RAT positivity at day five and seven found to be 22% and 6%, respectively. Another smaller study by Raza et al. which implemented a five-day isolation policy and deisolation following two consecutive negative RATs allowed 132 of 312 HCWs to return to work earlier than the previous 10-day isolation period [87]. In a South Korean study of viral kinetics in the Omicron era, onward rates of transmission were examined in HCWs exposed to others returning to work after five days of COVID-19 illness. The risk of onward transmission was low (4%) in the first five days following return; however, this study was conducted during a period of high community transmission and there were no corresponding WGS data available, thus limiting confidence in the exact source of transmission [109].

There is inherent variability in the duration of RAT positivity between individuals that should be considered when developing return-to-work policies. In contrast to Khan et al.’s RAT-positive rates of 22% and 6% at days five and seven, respectively, studies by Alshukairi et al. and Raza et al. demonstrated positive RAT rates of 45% at nine days and 36% at seven days [39,87]. RAT-positive rates as high as 96% at day five and 83% in day seven were described in a smaller study (n = 30) [110]. This variability suggests that testing-based return-to-work strategies may be more effective than blanket prescriptive deisolation periods which may expose patients to HCWs with delayed viral clearance times who may potentially transmit the virus [86]. However, despite this variability in viral shedding and RAT positivity, and even with blanket five-day isolation policies and no deisolation testing, the overall risk of contracting SARS-CoV-2 from returning HCWs remains very low [109]. Only 7% of 248 HCWs with a new diagnosis of COVID-19 were found to have any direct contact with returning HCWs, and only 4% had no other identifiable source of infection. This implies that the primary source of infection was likely to have been community contacts; it emphasises the importance of remaining at home and undergoing testing for respiratory viruses when unwell initially, but also supports the return to work when symptoms are improving after an appropriate time frame.

To our knowledge, there are no clear evidence-based recommendations for immunocompromised HCWs to return to work following a COVID-19 infection.

**Recommendation:** Following a recent COVID-19 infection, HCWs may return to work provided there is resolution of fever and acute respiratory symptoms and a negative RAT on day six post-symptom onset. Ongoing lethargy and a lingering dry cough may, however, be expected and should not preclude return to work. The use of surgical masks until at least day 10 post-infection is recommended to mitigate against the risk (albeit low) of onward transmission. Immunocompromised HCWs should ideally await resolution of fever and marked improvement in respiratory symptoms and two negative RATs or RT-PCR tests, taken on subsequent days, similar to immunocompromised patients, although this is a recommendation in the absence of clear evidence (Table 4).

Non-patient facing HCWs may return to work at an earlier time frame provided there is resolution of symptoms and a negative RAT; however, we would recommend ongoing mask use up to day seven post-symptom onset as a pragmatic risk-reduction measure, in line with evidence on recommended mask use [12].

## 8. Limitations

There are several limitations of note in our manuscript. Firstly, a narrative review has been provided, primarily to summarise the available literature thus far on an everyday issue that is heterogeneously approached by various infection and prevention control practitioners. Our research was limited to available full-text English-language papers and so may have excluded other important works, and owing to the breadth of the available literature, even with limiting search terms to a focus on ‘deisolation’, papers had to be manually screened for relevance, and so it is possible that important papers were missed; however, several authors independently reviewed the literature to minimise this risk. Secondly, further VOCs are likely to emerge as has even occurred during the process of manuscript preparation and revision, and likewise vaccination types, constituents, and schedules may all continue to develop, thus affecting the generalisability of these recommendations in the near future. The authors also hope that the approach to PPE choice and issues associated with inpatient isolation will continue to evolve. Rather than stringent recommendations to follow, we hope this narrative review provides a helpful working paradigm that can be adapted for use in individual facilities in light of future developments throughout the pandemic.

## 9. Conclusions and Future Directions

This narrative review has sought to summarise the available literature and guidelines examining SARS-CoV-2 transmission, viral kinetics, diagnostic methods, immunity, and inpatient deisolation following infection. The above recommendations are conservative given the consequences of onward transmission and the heterogeneity of available evidence at the time of writing. Individual facilities are recommended to adapt the framework presented above that involves a combination of disease severity, duration since symptom onset, underlying immune status, and availability of testing modalities in order to ultimately guide low-risk patient-centred deisolation.

Further research will clarify the impacts of several aspects affecting deisolation decisions. A more refined understanding of the transmission of SARS-CoV-2 IRPs in line with the new WHO definitions will guide the PPE required for the care of SARS-CoV-2-infected individuals or even of a recovering individual who may have prolonged viral shedding. As further VOCs emerge and while currently useful, RATs will need to be re-evaluated against RT-PCR and viral cultures for specificity in ruling out infectiousness. Guidelines ultimately need to provide a pragmatic solution that can be easily understood and adapted to staffing and bed allocation and ultimately that allow for ongoing patient care at the highest standard, and so future, prospective studies of deisolation protocols examining the R_0_ or onward infection rate would be best supplemented by qualitative insights into the patient and staff experience.

## Figures and Tables

**Table 1:** Severity of COVID-19 illness stratified according to symptoms, oxygen saturation, and chest imaging.

	Mild	Moderate	Severe
**Respiratory symptoms**	Symptomatic, no lower respiratory tract symptoms	Lower respiratory tract symptoms present	Lower respiratory tract symptoms present; often tachypneic > 30 breaths per minute
**Oxygen saturation**	Normal	Normal	<94%
**Chest imaging**	Normal	Abnormal	Abnormal, usually lung infiltrates > 50%

**Table 2:** Advantages and disadvantages of different SARS-CoV-2 testing modalities in deisolation protocols.

	Advantages for Use in Deisolation Protocols	Disadvantages for Use in Deisolation Protocols
**Real-time polymerase chain reaction (RT-PCR)**	Fast turnaround time, multiple targets, and likely to be adaptable to future variants	Unable to distinguish between non-viable or non-replicating virus; prolonged period of positivity especially if immunocompromised
**Rapid antigen detection (RAT)**	Especially rapid, inexpensive, point-of-care test. Reasonable specificity for transmissible virus	Analytical performance with future variants unknown; utility in cases of acute COVID-19 that are RT-PCR-positive but not RAT-positive is unclear
**Viral culture**	Gold standard. Especially helpful for immunocompromised patients	Labour- and time-intensive, generally only available in reference laboratories

**Table 3:** Select papers focusing on deisolation or progress testing, with positivity rates of various testing methods.

Paper	Population Assessed	Testing Method	Positivity (%) and Day (Post-Symptom Onset)	Comments
Khan et al. [86]	HCWs (n = 1216)	RAT	22% at day five 6% at day seven	Contributed 3799 additional days of work compared to 10-day isolation
Raza, Giri, and Basu [87]	HCWs (n = 240)	RAT	36% at day seven	HCWs required two negative RATs, 24 h apart, prior to returning to work
Alshukairi et al. [39]	HCWs (n = 480)	RAT	33% at day seven	All HCWs had received ≥2 doses of vaccination
Bouton et al. [88]	Patients (n = 92 for viral culture, n = 12 for RAT)	RAT Viral culture	RAT—75% at day five Culture—71% positive at day six	
Bays et al. [89]	HCWs (mathematical modelling of n = 500,000)	RAT	21% at day seven	Mathematical modelling
Mowrer et al. [90]	Hospitalised patients (severe or immunocompromised, n = 23)	RT-PCR	Ct value ≥ 30 for 84% between days 14–21	Percentages for Ct values ≥ 30; all patients PCR-positive at 21 days
			Ct value ≥ 30 for 100% by day 21	
La Scola et al. [35]	Hospitalised patients (n = 183)	Viral culture	Reportedly none positive after day eight	Culture negativity associated with RT-PCR Ct values ≥ 34
Van Kampen et al. [34]	Hospitalised patients (n = 129)	Viral culture	Mean duration of positivity is eight days (IQR 5–11 days)	

HCWs: Healthcare workers; RAT: rapid antigen test; RT-PCR: reverse transcription polymerase chain reaction; IQR: interquartile range; Ct: cycle threshold value.

**Table 4:** Summary of recommendations for deisolation or return to work.

COVID-19-Infected Individual	Immune Status	Disease Severity	Symptom Criteria	Testing Criteria	Release from Isolation or Return to Work	Comments
Patient	Immunocompetent	Asymptomatic, mild, or moderate	Asymptomatic by day five.	Negative RAT or RT-PCR test on day six or seven	Same day as negative RAT or RT-PCR test OR after day 10 without testing.	
Patient	Immunocompetent	Severe or critical disease	Resolution of fever and significant respiratory symptoms. Cough and lethargy may persist.	RAT or RT-PCR test on day 10	Following negative test on day 10 OR after day 20 without testing.	
Patient	Immunocompromised	Any	Resolution of fever and significant respiratory symptoms. Cough and lethargy may persist.	Testing to start on day 14 post-symptom onset; two negative RATs or RT-PCR tests, taken 24 h apart.	Following second negative RAT or RT-PCR test	Masks should be worn until at least day 20 post-symptom onset.
Healthcare worker	Immunocompetent	N/A	Resolution of fever and significant respiratory symptoms. Dry cough and lethargy should not preclude return to work.	Negative RAT (or RT-PCR test) on day six	Day seven	Wear surgical masks in patient-facing settings until day 10.
Healthcare worker	Immunocompromised	N/A	Resolution of fever and significant respiratory symptoms.	Two negative RATs (or RT-PCR tests) taken 24 h apart, first when clinically improved	Following second negative test	Consider non-patient facing alternatives to work if well but ongoing shedding.

RAT: rapid antigen test; RT-PCR: reverse transcription polymerase chain reaction.
